# Multimodal AI for Serious Illness Communication: Opportunities and Best Practices

**DOI:** 10.1093/geroni/igaf122.1468

**Published:** 2025-12-31

**Authors:** Ian Kwok, Kimberly Murdaugh

**Affiliations:** New York-Presbyterian Hospital/Weill Cornell Medicine, New York, New York, United States; New York-Presbyterian Hospital, New York, New York, United States

## Abstract

Patients and families face complex communication challenges in the setting of serious illness. These difficulties are compounded for individuals who are non-English-speaking, have low health literacy, have visual/auditory impairments, or possess unique learning styles. We hypothesize that augmenting standard verbal communication modalities with a multimodal communication approach (integrating written, visual, and other modalities) can improve medical information-sharing. In the first use of artificial intelligence (AI) for patient-facing serious illness communication, we developed Clinical SmartReporter™, a program capable of generating real-time transcribed reports of family meetings for patients, their caregivers, and other clinicians. Clinical SmartReporter™ uses machine learning, natural language processing, and speaker diarization modules to accurately integrate medical terminology, translate between multiple languages, and distinguish between a large number of different speakers. In this presentation, we will describe our model of multimodal communication, present results of our user-centered design process based on patient, caregiver, and clinician interviews, and demonstrate the use of Clinical SmartReporter™ in the setting of a simulated family meeting (professionally filmed video). We will outline technology-based opportunities for the advancement of communication science in palliative care research, quality improvement, and medical education. We will also facilitate a discussion of practical and ethical challenges, as well as the need to establish field-wide best practices for AI implementation in geriatrics and palliative care settings. These include data security considerations, partnership with institutional offices for technology licensing and privacy/security, and protection of the integrity of intimate patient-clinician relationships, emphasizing the need for clinician advocacy and cross-institutional collaboration.

